# Sera of elderly obstructive sleep apnea patients alter blood–brain barrier integrity in vitro: a pilot study

**DOI:** 10.1038/s41598-020-68374-8

**Published:** 2020-07-09

**Authors:** Anne-Cloé Voirin, Sébastien Celle, Nathalie Perek, Frédéric Roche

**Affiliations:** 1 Clinical Physiology, University Hospital (CHU), Saint-Étienne, France; 2 INSERM, SAINBIOSE U1059, UJM-Saint-Etienne, Université de Lyon, Saint-Étienne, France; 3 EA4607 SNA EPIS, Faculté de médecine Jacques Lisfranc, Université de Lyon, Campus Santé Innovation, 10 rue de la Marandière, 42270 Saint-Priest-en-Jarez, France

**Keywords:** Mechanisms of disease, Blood-brain barrier, Neurophysiology

## Abstract

Obstructive sleep apnea syndrome (OSAS) is characterized by repeated episodes of hypoxia during the night. The severity of the disorder can be evaluated using an apnea–hypopnea index (AHI). The physiological consequences are mainly cardiovascular and neuronal dysfunctions. One hypothesis to explain such associated neurological disorders is disruption of the blood–brain barrier (BBB), which protects the brain from endovascular cytotoxic compounds. We selected two subgroups of volunteers from the PROOF cohort study (France), a group of patients suffering newly diagnosed severe OSAS (AHI > 30/h) and a group showing no sleep apnea (AHI < 5/h). We exposed a human in vitro BBB model of endothelial cells (HBEC-5i) with sera of patients with and without OSAS. After exposure, we measured the apparent BBB permeability as well as tight junction and ABC transporter expression using whole cell ELISA. We showed that after incubation with sera from OSAS patients, there was a loss of integrity in the human in vitro BBB model; this was reflected by an increase in permeability (43%; *p* < 0.001) and correlated with a 50% and 40% decrease in tight junction protein expression of ZO-1 and claudin-5, respectively. At the same time, we observed an upregulation in Pgp protein expression (52%) and functionality, and a downregulation in BCRP expression (52%). Our results demonstrated that severe BBB disorder after exposure to sera from OSAS patients was reflected by an opening of the BBB.

## Introduction

Obstructive sleep apnea syndrome (OSAS) is a growing public healthcare issue in the world. It is a sleep disorder affecting 17–22% of the population aged 30–60 year^1^, with a high prevalence among the elderly and overweight subjects^2^. Upper airway collapse occurs repeatedly during sleep in such patients. This collapse involves many mechanisms such as intermittent hypoxia, activation of systemic inflammatory processes, and activation of the sympathetic nervous system. All these mechanisms lead to an increased risk of cerebrovascular and cardiovascular morbidity^3,4^. Recently, it has been shown that neurological consequences of obstructive sleep apnea (OSA) are similar to those found in Alzheimer's disease. Thus OSA would be one of the possible causes of Alzheimer's disease, with similar biomarkers for both diseases^5–7^. One hypothesis is that chronic intermittent hypoxia and sleep fragmentation might release several compounds inducing a neurotoxicity, which help to contribute to the progression of neurodegeneration. Nowadays, patients with OSAS are treated with a continuous positive pressure mask, and despite its effectiveness it remains sometimes poorly tolerated by patients^8,9^. This is why it is necessary to evaluate alternative or complementary therapies to limit these associated risks. A large number of studies focused their aim on the mechanisms linking sleep apnea to the deleterious effects of the cardiovascular system. Yet, cerebrovascular consequences of OSAS are less described and less understood. However, one hypothesis proposed to explain the mechanisms involved in sleep apnea is a disruption in one of the protective structures of the brain, i.e., the blood–brain barrier (BBB)^10^.

The BBB is a physical and metabolic barrier that separates blood flow from the central nervous system (CNS) compartment which regulates the passage of compounds and nutrients. This barrier would be disrupted under OSAS, leading to a cognitive deficit in patients associated with grey matter alteration**.** This barrier is composed of endothelial cells, astrocytes, pericytes, and neurons; with the whole representing the neurovascular unit^11,12^. Endothelial cells are the main cells that restrict and control the paracellular passage of compounds to the inside and outside of the brain. These properties are enabled by the presence of tight junctions (TJs) and transendothelial transporters such as ATP binding cassette (ABC) transporters^13^. The main transporters involved in transendothelial transport are energy-dependent and act as efflux transporters. They belong to the ABC transporter family, such as P-glycoprotein protein (Pgp) or breast cancer resistance protein (BCRP). In vitro BBB models are suitable to study BBB alteration under pathophysiological conditions. The ABC efflux proteins are essential for the barrier and particularly in the brain’s delivery of therapeutic drugs. Under a physiological state, the BBB is generally regarded like a mechanism which protects the brain. Nonetheless, in a number of pathologies, BBB disruption and change in ABC transporter expression have been suggested as a forecast factor in the progression of these diseases^14^. Loss of BBB integrity involves the brain being exposed to potentially cytotoxic concentrations of molecules due to the dysfunction of ABC transporters. This leakage into the circulation may disrupt brain homeostasis and adversely affect neuronal signaling.

We have developed in our laboratory a relevant human BBB model composed of an endothelial cell line (HBEC-5i)^15^, which is grown with a conditioned astrocyte culture medium without adjuvant. It has been shown that astrocytic cells have the ability to induce and maintain close junctions between endothelial cells^16,17^. This model can be used routinely in a laboratory. Our model presents many advantages such as stability during five days, which may allow are view of long term effects of therapeutic agents or physiopathological conditions. Our model could be used as a suitable tool in preclinical screening studies for assessing the permeability of human endothelial cells from the BBB.

In our clinical sleep research laboratory, we have set up a patient cohort composed of healthy elderly subjects (men or women; ≥ 65 years old), who had given their consent to participate in a clinical research program (Prognostic indicator of cardiovascular and cerebrovascular events (PROOF) cohort study). The whole population had been longitudinally evaluated using nocturnal polygraphy, allowing calculation of apnea plus hypopnea index (AHI), and a biological sample was collected to better evaluate blood biomarkers of sleep-related breathing disorders. From this cohort, we selected two groups of volunteers: a group of patients with severe OSAS, i.e., with an AHI > 30/h and a group without sleep apnea with an AHI < 5/h. These patients did not show other neurologic, cardiovascular or metabolic disorders other than OSAS for the severe OSAS group.

The aim of our present study was to evaluate the effect of sera from severe OSAS patients sera on an in vitro endothelial cell HBEC-5i model. The integrity of endothelial cells was assessed by apparent permeability measurements with sodium-fluorescein (Na-Fl)^18,19^ and expression of three TJ proteins (zonula occludens-1 (ZO-1), claudin-5 and occludin), that were well represented in the literature on BBB disruption in neurological disease^20^. Then, evaluation of transendothelial transport was also investigated for expression and activities of two efflux transporters (Pgp and BCRP). These two efflux pumps are particularly well represented in our model and were associated with cognitive deficit such as in Alzheimer’s disorders linked to BBB disruption^14,21^. Thus improved understanding of the molecular and cellular mechanisms that promote potential BBB disruption in OSAS, and a posteriori lead to CNS dysfunction (i.e., cognitive dysfunction, accelerated neurodegeneration), is required in order to develop therapeutic strategies.

## Results

### Subjects

We selected elderly patients with severe OSAS (AHI > 30) or no evidence of OSAS (normal: AHI < 5). The data collected for these subjects did not allow us to observe any difference, except for their AHI and gender which will be further discussed, their hypoxemic load, and their repeated autonomic activation during sleep (Table 1).

**Table 1:** Descriptive characteristics of the population selected for this study and from the PROOF cohort study.

Variables	Whole population	OSAS	No OSAS	*p*
Age (y)	75.9 ± 0.2	75.9 ± 0.4	75.9 ± 0.2	0.87
Sex (M/F)	15/14	11/3	4/11	< 0.001
AHI (h^−1^)	19.1 ± 3.2	38.9 ± 1.7	3.5 ± 0.4	< 0.001
ODI (h^−1^)	13.0 ± 2.4	24.4 ± 2.6	2.4 ± 0.5	< 0.001
SaO2 min (%)	88.4 ± 0.9	85.3 ± 1.4	91.3 ± 0.6	< 0.001
SaO2moy (%)	94.2 ± 0.3	93.6 ± 0.5	94.8 ± 0.3	0.04
%time SaO2 < 90%	4.4 ± 1.5	8.0 ± 2.5	1.1 ± 1.1	0.01
Total cholesterol (g L^−1^)	2.2 ± 0.1	2.2 ± 0.1	2.2 ± 0.1	0.9
HDL cholesterol (g L^−1^)	0.6 ± 0.0	0.6 ± 0.0	0.6 ± 0.0	0.8
LDL cholesterol (g L^−1^)	1.3 ± 0.1	1.3 ± 0.1	1.3 ± 0.1	0.9
Triglycerides (g L^−1^)	1.3 ± 0.1	1.3 ± 0.1	1.3 ± 0.2	0.9
Fasting blood glucose (g L^−1^)	1.0 ± 0.4	1.0 ± 0.5	1.0 ± 0.3	0.3
Hypertension medication (Y/N)	15/9	6/5	9/4	0.5
24-h mean SBP (mmHg)	117.6 ± 2.4	117.7 ± 3.4	117.5 ± 3.5	1.0
24-h mean DBP (mmHg)	72.2 ± 1.5	71.2 ± 1.8	73.2 ± 2.4	0.5

AHI: apnea plus hypopnea index; ODI: oxyhemoglobin desaturation index; SaO2: oxyhemoglobin saturation; HDL: high density lipoprotein; LDL: low density lipoprotein; SBP: systolic blood pressure; DBP: diastolic blood pressure.

### Impact of sera on HBEC-5i model

Optimal time and concentration of sera exposure have been previously validated to avoid cytotoxicity (data not shown). Our results showed that a 48-h incubation time and 10% sera concentration represented optimal culture conditions. These conditions were chosen for all of our subsequent experiments.

### Apparent permeability measurement

**Figure 1:**
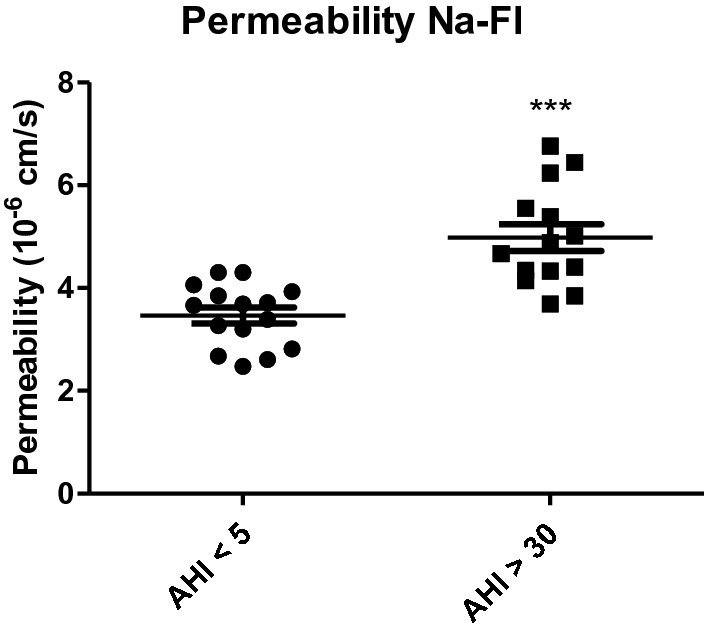
Membrane permeability measurement of HBEC-5i after 48 h of 10% sera from obstructive sleep apnea syndrome (OSAS) patients. OSAS patient AHI > 30 versus control sera AHI < 5. Results are represented as mean value ± s.e.m (n = 15). ***p < 0.001, AHI < 5 vs. AHI > 30. Na-Fl: sodium fluorescein; AHI: apnea–hypopnea index.

After 48 h of incubation with the sera, the apparent permeability measurement performed with Na-Fl showed a significant increase in Na-Fl permeability for severe OSAS patients (AHI > 30) compared to patients without OSAS (AHI < 5). We observed an increase in permeability coefficient from 3.46 (± 0.15) × 10^–6^ cm s^−1^ to 4.98 (± 0.26) × 10^–6^ cm s^−1^ (Fig. 1).

### TJ protein expression by whole cell ELISA

**Figure 2:**
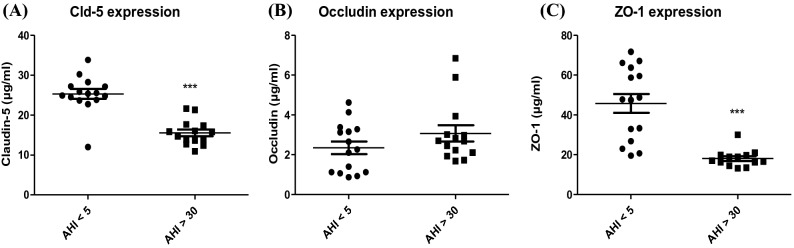
Expressions of Claudin-5 (**A**), occludin (**B**) and ZO-1 (**C**) after exposure of cells to 10% serum for 48 h. Results are represented as mean value ± s.e.m (n = 15). ***p < 0.001, AHI < 5 vs. AHI > 30. Differences in occludin expression between the two groups was not significant. AHI: apnea–hypopnea index.

Expression of TJ proteins (ZO-1, occludin, and claudin-5) were assessed after 48 h of exposure to sera from AHI < 5 versus serum from AHI > 30 (Fig. 2). Expression of ZO-1 and claudin-5 significantly decreased in OSAS patients with an AHI > 30 compared to patient with an AHI < 5 (all *p* = 0.001). Expressions decreased by 53% for ZO-1 and by 40% for claudin-5. There was no significant difference in the expression of occludin, which was 3.07 ± 0.4 µg mL^−1^ for AHI > 30 and 2.34 ± 0.31 µg mL^−1^ for AHI < 5.

### Impact of sera on transendothelial transport

#### ABC efflux transporters proteins (Pgp, BCRP) expression by whole cell ELISA

**Figure 3:**
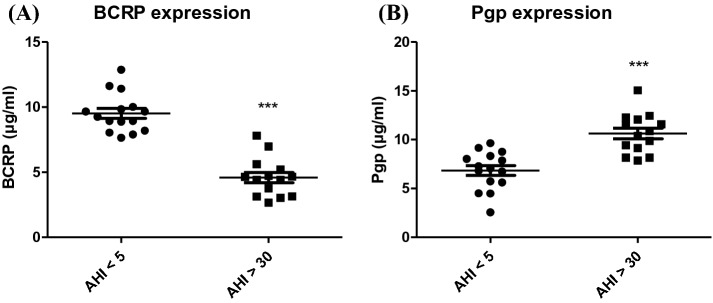
Expressions of BCRP (**A**) and Pgp5 (**B**) after exposure of cells to 10% serum for 48 h. Results are represented as mean value ± s.e.m (n = 15). ***p ≤ 0.001, AHI < 5 vs AHI > 30. AHI: apnea–hypopnea index.

Expression of ABC transporter proteins (BCRP and PgP) in our in vitro HBEC-5i model were evaluated after 48 h of exposure to sera from AHI < 5 versus sera from AHI > 30. PgP expression significantly increased by 52% with AHI > 30 compared to patients with AHI < 5 in contrast to BCRP expression, which significantly decreased by 52% for a serum-treated model of patients with AHI > 30 versus with AHI < 5 (Fig. 3).

#### ABC transporter functionality tests

**Figure 4:**
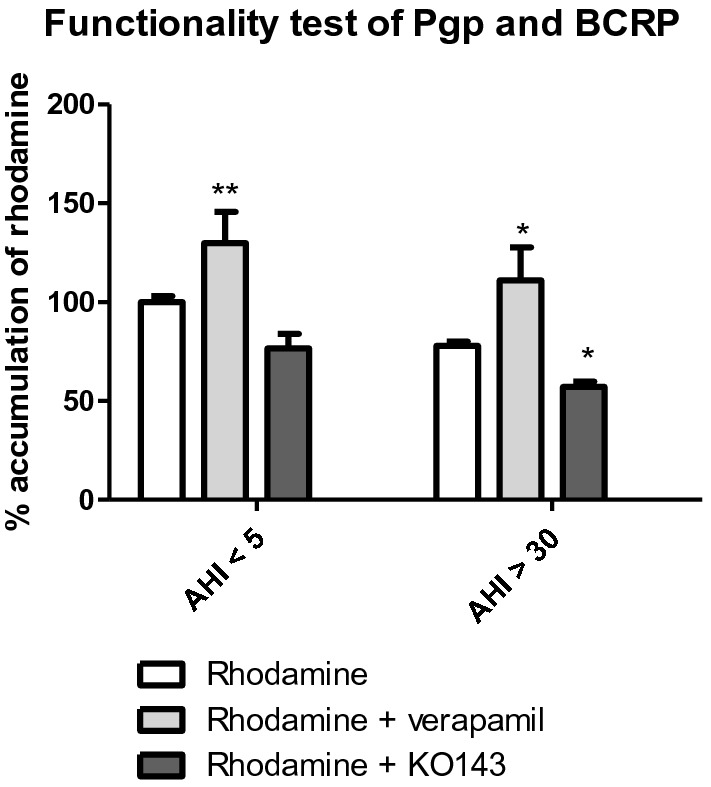
Functionality of rhodamine-123 transport by accumulation test with or without specific inhibitors of Pgp (verapamil) and BCRP (KO143) after exposure of cells to 10% serum for 48 h. Results are represented as mean value ± s.e.m (n = 15). **p < 0.01 between rhodamine and rhodamine + verapamil, *p < 0.05 rhodamine versus rhodamine + verapamil or KO143. AHI: apnea–hypopnea index.

Functionality of ABC transporters were evaluated by studying a fluorescent substrate (rhodamine-123) transport of the ABC efflux transporters (PgP and BCRP). We studied the accumulation rate (i.e., a percentage of accumulation) of this substrate within cells with or without Pgp or BCRP specific competitive inhibitors, i.e., verapamil and KO143, respectively. First we observed a tendency for patients with sleep apnea (AHI > 30) to have a decrease of 23% in rhodamine-123 accumulation (*p* = 0.0571), suggesting that ABC efflux was more functional in severe OSA patients. In order to see which efflux pump, Pgp or BCRP, was most involved, we used specific competitive inhibitors. In the presence of verapamil, the competitive Pgp inhibitor, we observed an increase of 55% and 40% in rhodamine-123 accumulation for patients with AHI < 5 and AHI > 30, respectively, indicating an important activity of the Pgp efflux pump. In contrast, the use of KO143, a BCRP-specific inhibitor, showed a decrease of 43% in accumulation of rhodamine-123 that was related to the under expression of this protein in AHI patients > 30 (Fig. 4). All these results therefore showed a positive regulation of Pgp, with a more important activity in endothelial cells incubated with sera from severe OSAS patients.

## Discussion

OSA is often associated with neurological deleterious consequences in the elderly population. One of the hypotheses put forward by Lim and Pack consists of a BBB disruption provoked by several neurotoxic compounds released and circulated in blood as a consequence of intermittent hypoxia, sleep fragmentation, and also by associated inflammation^22^ or an associated metabolic disorder. However, today there is evidence of this hypothesis regarding the alteration of the BBB following an OSA. Indeed, the imaging data showed that brain regions near the hippocampus had a volume that could either be increased or decreased. The explanation for an increased volume would be inflammation and activation of glial cells, while a decrease would suggest cell loss leading to neurodegenerative damage^23^. In addition, other studies described a decrease in OSA patients in entropy measurement that indicates an acute change in cerebral tissue and links it to the rupture of the BBB^24,25^.

However, the BBB is an essential structure for the protection of the CNS, limiting exchanges between the blood circulation and the brain through transporters and TJ proteins^26,27^. TJ proteins act as a physical barrier that regulates paracellular transport; this specialized phenotype is fundamental for the protection of the brain. Moreover, efflux ABC family pumps are a protective mechanism that actively efflux compounds outside the endothelial cell and hence inside the bloodstream. These proteins act like a transendothelial barrier that reduces the exposure of the CNS to xenobiotics. Since the structural and functional integrity of transporters and TJ proteins are necessary for an intact BBB, alteration of these components is a key event in the BBB’s impairment. Moreover, in several pathologies it has been shown that a dysregulation of ABC transporters such as Pgp and BCRP contribute to a pharmaco-resistant phenotype as in Alzheimer’s disease and epilepsy^28,29^.

Nowadays, patients with severe OSAS are mainly treated with a continuous positive pressure airway pressure that remains sometimes poorly tolerated by patients^9^. It is crucial to find therapies to limit neurological associated risks. However, there are no predictive tests to evaluate deleterious effect of sleep apnea on the integrity of the BBB and more precisely on endothelial cells that are a key element to form the protective barrier. In our laboratory, we have developed a human BBB model, composed of HBEC-5i endothelial cells, in cooperation with conditioned media from human astrocytes. In this study, we have screened the effect of sera from patients on TJ proteins and ABC transporters.

We have evaluated the effect of sera on TJ proteins and ABC transporters. Sera from patients were selected with and without OSAS from the PROOF cohort, a cohort with an extensive phenotype evaluation of sleep disorders. This is the first time that a screening of sleep apnea sera from patient effects has been realized on such an endothelial model of human BBB. This work shows that the HBEC-5i model may be used as a predictive model of cerebrovascular consequences related to the release of cytotoxic compounds in many diseases. Our results showed a significant increase in the apparent permeability following exposure with sera (Fig. 2), which showed that the BBB lost some of its physical properties and that small molecules such as Na-Fl can cross the cell barrier. This loss of permeability was evaluated by the expression of TJ proteins, since they are responsible for the physical characteristics of the BBB. Sera from patients with AHI > 30 significantly decreased the expression of ZO-1 and claudin-5 proteins, while occludin expression remained unchanged (Fig. 3). ZO-1 and claudin-5 being the main actors in the development and maintenance of the BBB^30^, and a change in their expression or location led to a dysfunction of the BBB. Nevertheless, in accordance with other studies, our work showed that occludin does not play a major role in maintaining the barrier but is linked to the regulation of calcium through the BBB^31^. Prior studies have demonstrated a loss of expression of ZO-1 in in vitro human and mouse models examining the effects of amyloid beta protein on the BBB, suggesting that this process may occur in Alzheimer's disease^32,33^. Therefore, our results showing modification of the expression of the TJs are similar to those prior findings which implied loss of permeability in neurodegenerative disease^32,34^. The role of claudin-5 in such BBB dysfunction has been less recognized, but alteration of such TJ protein expression could be also interpreted as a complementary frailty of the physical properties of the barrier^35^.

In a second part, we evaluated the effect of sera on efflux pumps (ABC transporters), which efflux potentially cytotoxic compounds. These transporters are dysregulated in case of neurodegenerative disease. We showed an increase in Pgp and a decrease in BCRP expression (Fig. 4), which are data found in the literature, especially during an inflammatory stress process^36^ or after intermittent hypoxia stress. The activity of efflux pumps, evaluated from the accumulation of a fluorescent substrate, showed that sleep apnea patient serum provoked an increase in ABC efflux pump activity. The results obtained after competitive inhibition is in line with a Pgp overexpression and functionality rather than BCRP protein. BCRP is under expressed^36–39^.

Few studies until now have worked on the possibility to evaluate the effect of sera from patients on endothelial cell openings and the functionality of their ABC transporters. Our results are important as it has demonstrated that sera from OSAS patients affect endothelial cells and many mechanisms seem to be involved to induce a BBB breakdown. One working hypothesis is the systemic inflammation in a chronic hypoxic setting. Indeed, a link has been shown between systemic inflammation and OSA, with an increase in pro-inflammatory cytokines during sleep apnea^40–42^. Nadeem et al. and others showed a higher pro-inflammatory cytokine concentration for patients with sleep apnea syndrome^41,43^. This inflammation is known to have a deleterious effect on the integrity of the BBB, and it plays a role in ABC transporter regulation^36,44,45^. However, such pro-inflammatory interleukin activation is not constant in OSAS patients and more particularly in adults and in the elderly. Furthermore, microparticles described in OSAS sera patients could lead by themselves to endothelial dysfunction at the brain vascular scale and could alter BBB permeability, like as found in an exosome^46,47^. Indeed, serum factors are not yet identified, and inflammation remains a hypothesis. Other pathways such as excessive vessel shear stress could also be involved in disruption of the BBB^48^. The discovery of the opening of the BBB after sera exposure suggests that this opening may be partly responsible for cognitive deficits; but another track of exploration is that fluid movements generated by the opening of this barrier could be involved in some cases of increased intracranial pressure. In this sense, it would be interesting to study the entry and flow processes of cephalo rachidien liquid, including alterations in aquaporins^49^. Thus, the different pathways involved in OSAS leads to the release of many molecules such as interleukins, VEGF, elements of the oxidative stress pathway, or exosome vesicles that could be found in sera; and it would be interesting to see if these hypotheses are true in our sera of elderly patients.

Our study has some limitations. First of all, the “human” in vivo BBB function in OSAS is not well (and not easily) evaluated. Our observations on a BBB in vitro model could not be directly extrapolated. However, several authors using brain MRI methods proposed an alteration of the BBB function in OSAS with an increased in permeability of the barrier in hypoxic conditions^50^. The impact of OSAS on brain volumes in several critical brain areas is highly probable according to VBM methodology, and astrocyte pre-activation has to also be demonstrated in a mouse model of chronic intermittent hypoxic exposure, suggesting a deleterious cellular message coming from the intravascular compartment in OSAS. Secondly, the concentration of sera in the cell culture nutritive fluid could not represent the real concentration of cytotoxic components in patients suffering OSAS. In this study, only 10% was mixed with medium (without fetal bovine serum^51^), but in physiological condition we would find 100% of the sesera’s components. Moreover, exposure to repeated hypoxemic stress could lead to a chronic adaptation of the “physiological” endothelial cells which limit the permeability. We could underline here that we tested a short exposure to sera components. The model used has a permeability plateau during which experiments are possible and which limits the possible duration of exposure. However, our results demonstrated a deleterious impact of sera of severe OSAS on endothelial function. Thirdly, there are biases in the selection of patients in our preliminary study. We used only an elderly patient population, which are susceptible to more comorbidity factors. Despite the use of an elderly population, which has a higher prevalence for this syndrome, the study of a younger population would also bring its share of knowledge.

In addition, the male:female ratio in our study is a bias. Indeed there are more males than females with OSAS. This can lead to an interpretation bias and is explained by the fact that diagnoses in females are often under evaluated^52^ and that the prevalence of OSAS is higher in males^53^. Nevertheless, studies have shown a gender influence, such as on hippocampal volume in OSAS^23^ or greater cortical lesions in women with OSAS^54^. Consequently, sex ratio is a point that will have to be taken into account later on.

## Conclusion

In conclusion, we demonstrated for the first time that blood compounds from severe OSAS patients induced an endothelial stress, which led to a BBB’s disorder, reflected by an opening of the paracellular pathway, with a possibility for cytotoxic compounds crossing the barrier. In response to the paracellular dysfunction, endothelial cells increased efflux transport to fight against the entry of possible cytotoxic compounds into the brain and/or to accelerate the return or the efflux of intracellular cytotoxic/neurotoxic components into the vascular flow. This study was conducted on an in vitro model of culture of cerebral endothelial cells in conditioned media. This model could be improved with astrocytes and pericytes; nevertheless, it has been shown earlier that it is a good pharmacological model^15^. Our work showed that our model may screen patient serum and may be an interesting tool to evaluate risk and may be, one day, the reversal effects in sleep apnea patients. It is important to identify OSAS patients when assessing patients with depression or cognitive impairment. Therapeutic treatment of OSAS might change the deteriorating trajectory of elderly patients with already diagnosed vascular depression and cognitive impairment/dementia. Moreover, our model may be used to predict BBB permeability in other sleep-related disorders.

## Materials and methods

### Subjects

Sera from patients had been obtained from the PROOF cohort study. The PROOF recruitment consisted of 1,011 volunteers aged ≥ 65 years and living in Saint-Etienne (France). The methods of evaluation and follow-up of the PROOF study have been described in laboratory studies^55^. In this cohort, sera from 29 subjects were selected: 14 subjects with severe untreated (newly diagnosed) OSAS (AHI > 30) and 15 patients without OSAS (AHI < 5).

The local Ethics Committee and the University Hospital (CCPRB Rhone-Alpes Loire: IRBN532016 and IRB2002/22/CHUSTE) approved the PROOF Study. The CNIL (national committee for information and liberty) gave agreement at this time for data collection. We confirm that all research was performed in accordance with guidelines and regulation.

### Consent statements

All subjects gave their written consent to participate in the PROOF study. Blood was collected the morning after polygraphic recording and frozen samples had been anonymous, in accordance with good practice. Sera samples were frozen at − 80 °C and conserved in such conditions in our sera collection. All subjects gave their informed consent to participate in this study. Clinical trial numbers of PROOF and SYNAPSE studies are NCT00759304 and NCT00766584.

### Chemicals and reagents

Astrocyte cells (human astrocytes) were from ScienCell Research Laboratory (Carlsbad, CA, USA) and endothelial cells (HBEC-5i, human brain endothelial cells) were obtained from ATCC (Manassas, VA, USA). Specific inserts for 24-well (transparent PET membrane, 0.45-µm pore diameter size) were from D. Dutscher (Alsace, France). EVOM voltohmmeter system was purchased from World Precision Instruments (Hertfondsire, UK). Occludin antibody was from Life Technologies (Saint Aubin, France), and ZO-1 was from GeneTex (San Antonio, TX, USA). Pgp, claudin-5, BCRP and MRP-1 antibodies came from Santa Cruz Biotechnology (Dallas, TX, USA). All compounds for Ringer HEPES buffer were from Sigma-Aldrich (St Quentin Fallavier, France) as well as BCECF-AM, verapamil, KO143, probenecid, Na-Fl, DMEM-F12 (Dulbecco’s Modified Eagle’s Medium) and methyl-thiazolyl-tetrazolium (MTT). Rhodamine-123 was from Thermo Fisher Scientific (Waltham, MA, USA).

### In vitro blood–brain barriermodel used

**Figure 5:**
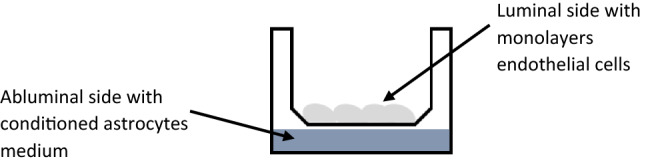
Schema of the human in vitro model.

The BBB model was composed of HBEC-5i endothelial cells and was cultivated with human astrocyte conditioned medium on insert as previously described^15^. All cells were cultured in DMEM-F12 HAM and used before passage number 20, which is the time where these cells may begin to lose their BBB properties. HBEC-5i cells were seeded onto the luminal side of the insert at a density of 4 × 10^5^ cells cm^2−1^ and cocultured with conditioned medium for 14 d, the time to necessary to reach optimal permeability (Papp), and it was maintained and stable for 5 d in this model as shown in Fig. 5.

### Sodium-fluorescein permeability measurements

The paracellular barrier function of the endothelial cells was evaluated by measuring apparent permeability of cells to sodium-fluorescein (Na-Fl), which is a hydrophilic fluorescent molecule (MW: 376 Da). Firstly, the cells were washed with Ringer HEPES buffer, then Na-Fl was diluted to 10 µg mL^−1^ with Ringer HEPES buffer. It was placed onto the luminal side of the insert and left at 37 °C for 1 h. After 1 h, samples were removed from the abluminal side and replaced with Ringer HEPES buffer. The Na-Fl concentration was obtained using a fluorescence spectrophotometer (FluoroskanAscent™, Thermo Fisher Scientific, France), with excitation (485-nm) and emission (530-nm) wavelengths. Papp is measured in cm s^−1^ and is calculated using the following formula:

$$P_{app} = \frac{V_r}{{C_o}} \times \frac{1}{S} \times \frac{C_1}{t}$$

where P_*app*_ is the apparent permeability, V_r_ is the volume of medium in the abluminal side, C_0_ and C_1_ are the concentration of fluorescent compound in the luminal chamber at t_0_ and in the abluminal side after t time, i.e., 1 h of incubation, and S is the monolayer’s area^56^.

### Whole cell ELISA assay

Inserts with HBEC-5i monoculture were fixed for 20 min with 4% para formaldehyde at room temperature, before cells were washed with 1% BSA diluted in PBS at pH 7.4. After fixation, a blockade of the endogenous peroxidase site was performed for 20 min with 3% H_2_O_2_ diluted in methanol. This was followed by a blocking of unspecific staining with 20% normal goat serum. Cells were incubated with monoclonal mouse anti-Pgp (2 µg mL^−1^), rabbit anti-ZO-1 (4 µg mL^−1^), rabbit anti-occludin (1 µg mL)^−1^, rabbit anti-BCRP (2 µg mL^−1^) or rabbit anti-claudin-5 (2 µg mL^−1^) antibodies, respectively. Then cells were washed and incubated with secondary antibody peroxidase conjugated anti-mouse or rabbit IgG for 2 h at room temperature (diluted at 1/750). After cells were washed, TMB substrate was added for 10 min at room temperature and in the dark. After HCl neutralization, the reaction product color reagent was measured at 490-nm with a spectrophotometer.

### Drug transporter activity assays

Transendothelial transport activity was measured in assessing the transport of specific substrates, i.e., rhodamine-123 for BCRP and Pgp, in the presence and absence of competitive inhibitors such as verapamil for Pgp and KO143 for BCRP. Cells were cultured in specific DMEM and washed, then cells were pre-incubated with or without inhibitors for 15 min at 37 °C. Inhibitors (100 µM) were added in the luminal side to study the transport from the luminal to abluminal side and conversely. The luminal compartment was incubated with rhodamine-123 (1 mM) for 1 h at 37 °C. Finally, cells were lysed with 1% SDS, and fluorescence was obtained through a fluorescence spectrophotometer at 493-nm (excitation) and 515-nm (emission) wavelengths.

### Statistical analysis

Statistical analysis was realized using Mann–Whitney test. GraphPad software was used for statistical analysis. The differences between means were considered to be significant when *p *values were < 0.05, and the value was expressed as the mean ± s.e.m. Except for Table 1, statistical analysis was done with Stata 11 software and a t-test.
